# Beyond Placement of Pedicle Screws - New Applications for Robotics in Spine Surgery: A Multi-Surgeon, Single-Institution Experience

**DOI:** 10.3389/fsurg.2022.889906

**Published:** 2022-06-16

**Authors:** Troy Q. Tabarestani, David Sykes, Kelly R. Murphy, Timothy Y. Wang, Christopher I. Shaffrey, C. Rory Goodwin, Phillip Horne, Khoi D. Than, Muhammad M. Abd-El-Barr

**Affiliations:** ^1^ Duke University School of Medicine, Duke University Hospital, Durham, Durham, NC; ^2^ Department of Neurosurgery, Duke University Hospital, Durham, Durham, NC; ^3^ Department of Orthopedic Surgery, Duke University Hospital, Durham, Durham, NC

**Keywords:** robotic-assisted, spine surgery, neurosurgery, kambin’s triangle, pedicle screw, percLIF, iliac screw, sacroiliac joint fusion

## Abstract

Interest in robotic-assisted spine surgery has grown as surgeon comfort and technology has evolved to maximize benefits of time saving and precision. However, the Food and Drug Administration (FDA) has currently only approved robotics to assist in determining the ideal trajectory for pedicle screw placement after extensive research supporting its efficacy and efficiency. To be considered a durable and effective option, robotics need to expand beyond the indication of just placing pedicle screws. This article aims to illustrate a multi-surgeon, single-institution experience with unique applications of robotic technologies in spine surgery. We will explore accessing Kambin’s Triangle in percutaneous transforaminal interbody fusion (percLIF), iliac fixation in metastatic cancer, and sacroiliac (SI) fusions. Each of these topics will be covered in depth with associated background information and subsequent discussion. We show that with proper understanding of its limitations, robots can help surgeons perform difficult surgeries in a safe manner.

## Introduction

From 2012 to 2018, the use of robotic-assisted (RA) surgeries has increased in incidence from 1.8% to almost 15% for all general surgeries (1). Specifically in the realm of spine surgery, RA procedures have been examined extensively for use in pedicle screw placement during minimally-invasive spine surgery (2, 3). With decreased muscle retraction and dissection, RA screw placement has not only improved post-operative outcomes for patients but has also assisted with the safety and accuracy of pedicle screw placement (4–6). With the combination of 3-dimensional (3D) imaging techniques, higher resolution MRIs, and more advanced robotic function, RA surgeries are primed for a rapid expansion throughout the field.

One of the main advantages of RA surgeries is the ability of the robotic arm to guide the surgeon to a predefined location in 3D space and allow for specific trajectories. The application of this ability to place pedicle screws under image navigation is well-studied in the current literature (7). However, as surgeons have become more comfortable with robotic assisted surgery, they have begun using the robot for other ‘off-label’ applications such as planning and executing osteotomies, decompressions, and interbody fusions (8–10).

In this paper, we review a multi-surgeon, single-institution experience of unique applications of robotics as it pertains to spine surgery. We aim to exemplify three types of procedures to illustrate the expanded applications of RA surgeries: (1) accessing Kambin’s Triangle in percutaneous lumbar interbody fusion (percLIF), (2) percutaneous iliac screw fixation, and (3) sacroiliac (SI) joint fusions. We highlight the advantages and disadvantages of robotic assistance in these cases, discuss their prevalence in the literature, and speak on the future directions for RA surgeries.

### Application 1: Robotic-Assisted Access into Kambin’s Triangle During percLIF

A procedure that has gained popularity in the care of patients with degenerative spondylolisthesis or disc disease is percLIF through Kambin’s triangle (11, 12). Kambin’s triangle is defined as the exiting nerve root, superior endplate of the caudal vertebral body, and superior articulating process (SAP) (13). The triangle allows surgeons to avoid a facetectomy when attempting to access the disc space, however, the space has wide variety depending on the level, ranging from 60 mm^2^ at levels L1-L2 to 108 mm^2^ at levels L4-L5 (13–15). Traditionally, biplanar fluoroscopy is used for localization in spine surgery, but its application might be limited in smaller areas such as Kambin’s triangle. We have recently shown that robotic entrance into the disc space and interbody placement is a feasible alternative as demonstrated in a single-center, retrospective review of ten patients with spondylolisthesis who underwent RA percLIF using robot-guided trajectory to access Kambin’s triangle for cage placement (16).

**Figure 1:**
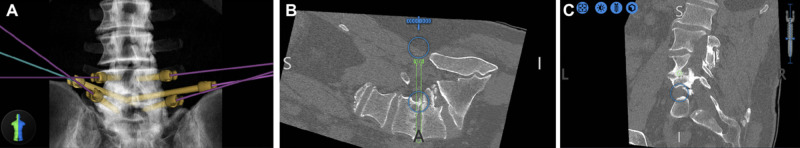
Preoperative plan (**A**) Pedicle screws and bilateral projections into Kambin’s triangle were planned. (**B**) The right projection is highlighted in green. (**C**) Coronal plan shows entrance into disc at the mid-pedicle point, which is the largest area of the safe zone within Kambin’s triangle (from (16), used with permission).

In these cases, the robot utilized CT anatomy to plan a trajectory into the intervertebral space through Kambin’s triangle (Figure 1). As this trajectory enters the disc space from the inferior-most corridor through Kambin’s triangle, traversing neural anatomy is safely avoided. The trajectory is then backpropagated to the skin to ensure no structures would impede entrance into the disc space. Using the robotic arm as a guide, a stab incision is made in the skin and dilators were used to widen the fascia and subcutaneous tissue until right before entering Kambin’s triangle. Then, a k-wire is passed through the end effector of the robot and entered the disc space through Kambin’s triangle. The k-wire is maintained, and the robot is moved out of the way. Further dilation is done until an 8 mm portal is placed, followed by adequate discectomy. Adequate discectomy was confirmed by inserting a balloon into the disc space and filling it with contrast agent which could subsequently be visualized to confirm contact with the inferior endplate of the superior vertebral body and the superior endplate of the inferior vertebral body. A k-wire was again placed through Kambin’s triangle which was used to guide an obturator into the disc space. Bone morphogenetic protein (BMP) was placed in the anterior disc space, followed by implantation of the ELITE (Spineology, Minneapolis, MN) expandable interbody fusion device.

This study showed an average estimated blood loss with 68 mL compared to average ranges of 360–7,000 mL for instrumented fusions (17). For the post-operative results, no patients were lost to follow-up, no patients were readmitted, and disc heights for all the patients showed statistically significant increases with the expandable cage. More importantly, no patients experienced post-operative motor or sensory deficits. To add to the impressive precision of this RA technique, each of the patients had a smaller Kambin’s triangle than the average typically reported size. Patients who underwent RA percLIF were discharged on average after 1.8 days, which compares favorably to an average LOS of 3.6 days for elective spine fusion (18). The LOS of 1.8 days remained lower in comparison to other minimally-invasive spine surgery LOS averages (19). Without the need for laminectomies or facetectomies, minimal tissue disruption was attained by using RA instrumentation finely tuned to each patient’s unique spinal landscape.

### Application 2: Robotic-Assisted Percutaneous Iliac Screw Fixation

**Figure 2:**
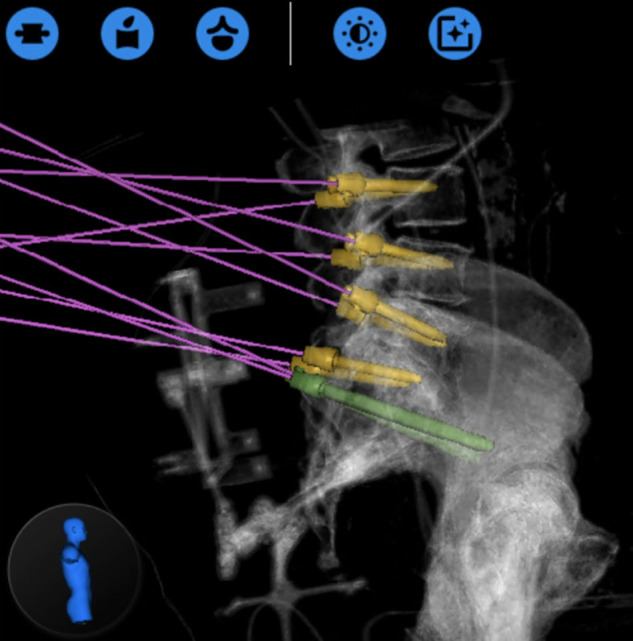
Globus ExcelsiusGPS® preoperative planning phase showing the placement of the iliac screws prior to their insertion (from (22), used with permission).

**Figure 3:**
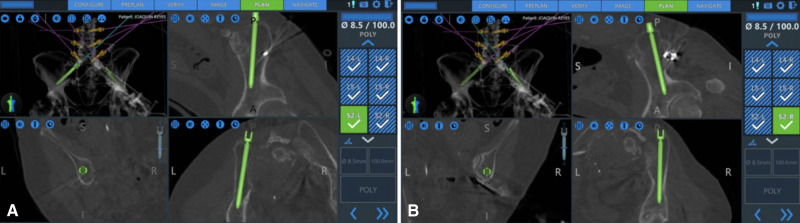
Navigated placements of the right (**A**) and left (**B**) iliac bolts (8.5 × 100 mm) (from (22), used with permission).

Robotic assistance can also be useful for iliac screw fixation in the presence of destructive lesions of the pelvis and sacrum. Over the past years, there has been a general trend away from the Galveston rod technique/iliac screws and towards the S2-alar-iliac screw (S2AI) (20). The S2AI screw provides similar stability for the patient without the need for side connectors. However, in the presence of a more destructive and expansive sacral lesion, S2-AI screws may not be optimal (21). We have recently published our early experience of RA iliac fixation for patients with destructive sacral lesions (22). For the two cases presented here, both patients underwent percutaneous iliac screw fixation without the need for side connectors. RA navigation allowed the surgeon to plan a modified screw trajectory and entry site in line with the lumbar pedicle screws under the skin, eliminating the need for 3-D rod contouring or connectors (Figures 2, 3).

**Figure 4:**
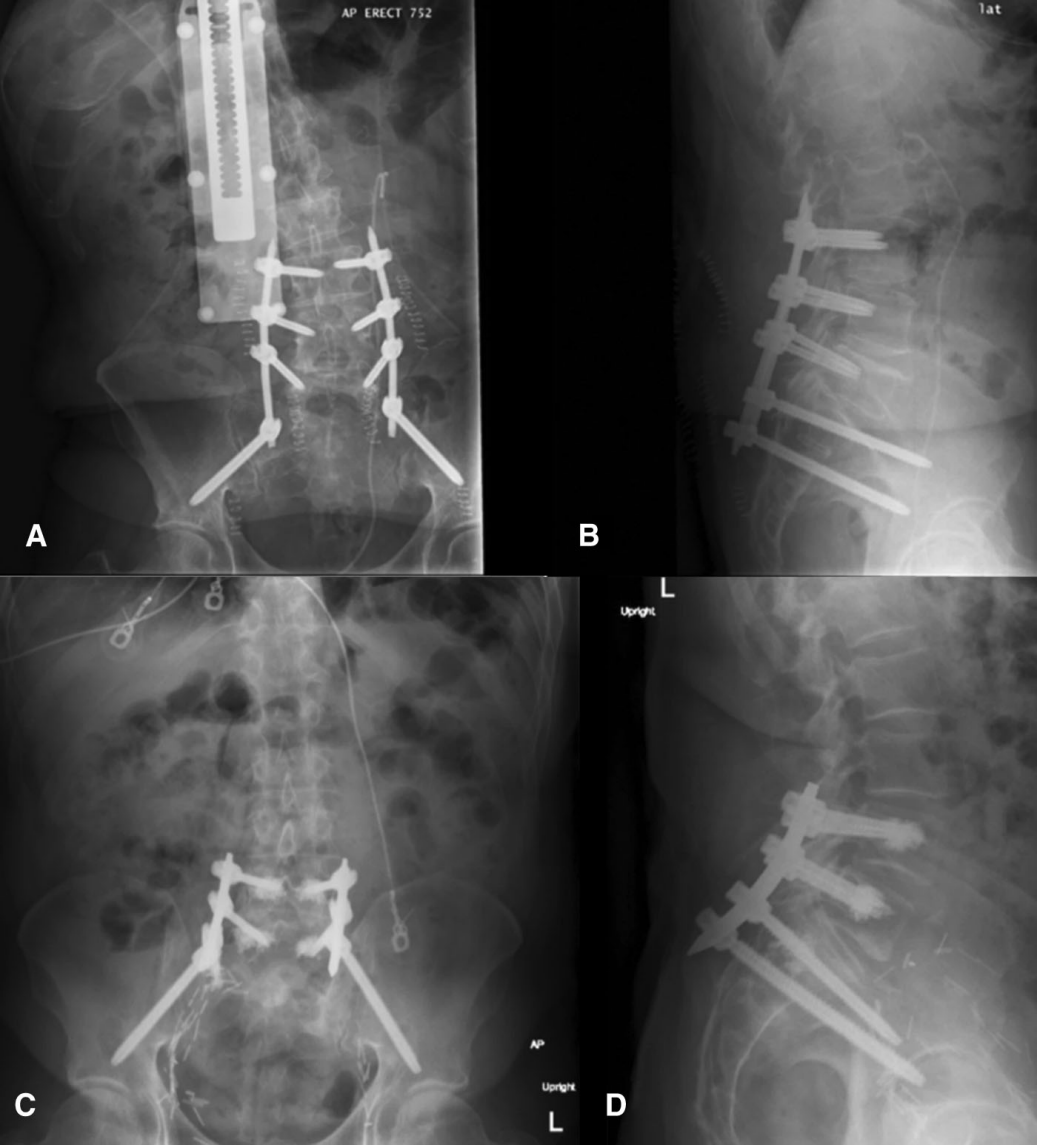
Postoperative upright x-rays for Cases 1 ((**A**) AP; (**B**) lateral) and 2 ((**C**) AP; (**D**) lateral) of the fixation construct demonstrating good screw placement (from (22), used with permission).

The operative plan was very similar in both cases: intraoperative anterior–posterior and lateral fluoroscopic images of L4-S2 were merged with preoperative CT, Globus ExcelsiusGPS® was positioned correctly, and the screws were placed. Bilateral fluoroscopic “teardrop/outlet-oblique” views and an intraoperative CT confirmed proper alignment and placement of all screws (Figure 4); however, the screw positions were not assessed using any form of grading system. Neither patient had post-operative complications or instrumentation failure at their respective follow-ups.

In both cases presented, the use of S2AI screws was precluded by the extensive damage to the sacrum. Therefore, a modified iliac screw entry point was utilized with entry point slightly more medial than the traditional method, with a pre-planned trajectory of the screws using robot assistance (23, 24).

### Application 3: Robotic-Assisted SI Joint Fusion

Open SI joint fusions began in the 1920s, progressed with sacroiliac screw fixations in the 1980s, and eventually joined the minimally invasive field in 2004 (25–28). Prior to this hallmark, there were not many reports of standalone SI joint fusions, but rather mentions of them in larger instrumented spine procedures. While there was still doubt regarding the long-term efficacy, randomized clinical trials and patient follow-up studies began revealing that minimally invasive SI fusion surgery was safer and more efficacious than the usual conservative management (29, 30). As with the other minimally invasive surgeries, the fusions showed promise by reducing operative time, blood loss, and LOS (31). Even though short-term fusion rates were low, long-term radiographic analysis revealed high rate of successful bone apposition to implants (32).

The current literature supports two main approaches for SI joint fusion: dorsal or lateral transarticular, with the latter being more commonly used. In the lateral technique, screws are packed with graft materials to promote bony growth across the joint. In the dorsal approach, the implant is placed obliquely through the SI joint space (33). Both techniques have shown to be equally beneficial for post-operative fusion rates and range of motion (34). However, just as with the other techniques described, robotic assistance for SI joint fusion has yet to be fully documented in the literature. In 2021, Piche et al. provided the first known technical guide with specific case reports to describe their methodology (35). In 2022, our group at Duke University published a case series displaying this “off-label” use of the robot implementing a similar surgical technique (36).

**Figure 5:**
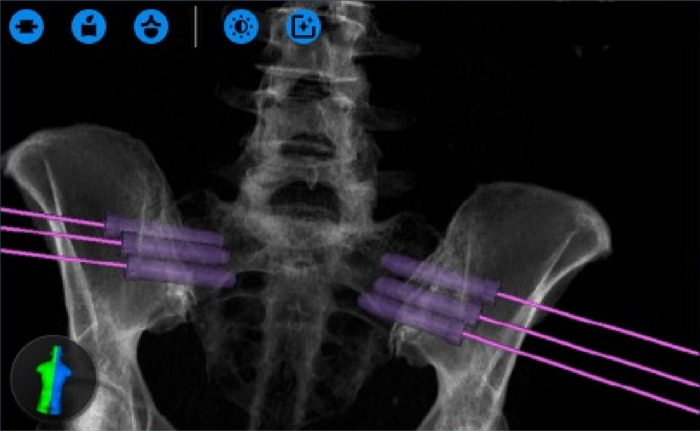
Screw entrance plans designed on the ExcelsiusGPS interface, which requires bilateral trajectories to be mapped (from (36), used with permission).

**Figure 6:**
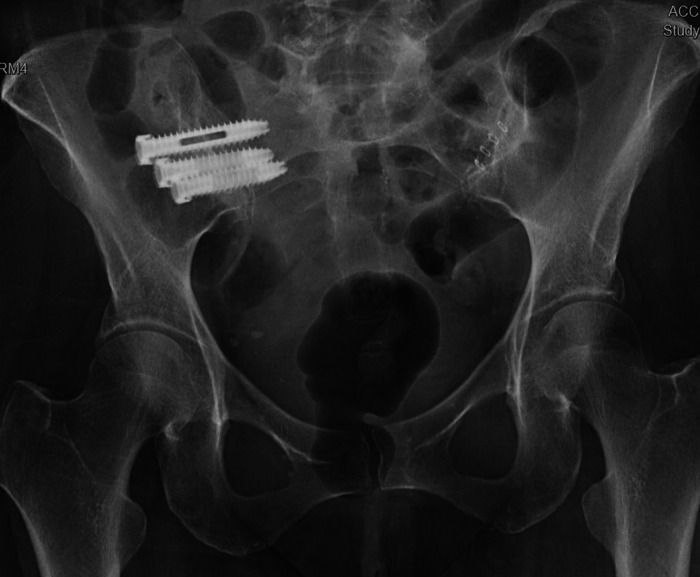
Post-implantation X-rays demonstrating appropriate placement of the hydroxyapatite-coated titanium implants through the right SI joint (from (36), used with permission).

Across all 9 patients in the cohort, three implants were planned with trajectories designed to traverse the SI joint (Figure 5). After general anesthesia was obtained, dynamic reference bases were placed in the contralateral posterior superior iliac crest. Anterior-posterior and lateral x-rays were then taken of the L5 vertebral body and sacrum, which were merged with a preoperative CT scan containing the pre-planned trajectories. Once the surgeon confirmed the image fusion accuracy, the robot was wheeled into the operative field until the end-effector could reach the entire work zone. The end effector was placed in the starting location and, using the robot as a guide, a high-speed drill burred a pathway for the SI screws following each respective trajectory. Intraoperatively, three screws (one above, the second short, and the third below the S1 foramen) were placed under the navigational guidance of the Globus robot. No EMG monitoring leads were placed during the procedure, but confirmatory X-rays were taken to ensure the screws remained lateral to the sacral foramina and superior to the acetabulum (Figure 6). Post-operatively, the patients were immediately allowed to weight-bear as-tolerated. The average operative time was 55 min, which decreased over the course of this case series. The average intraoperative radiation exposure was 13.2 mGy, average length of stay was 0.4 days, and there were no intraoperative complications or conversions.

## Discussion

The history of robotics, as it relates to spine surgery, extends back almost 30 years. Since then, the dual excitement for both minimally invasive surgeries (MIS) and robotics has grown exponentially together for a multitude of reasons. As demonstrated through the applications reviewed in this article, the combination of MIS and robotic assistance into one procedure can improve various outcomes for the patient and surgeon alike. With its ability to precisely guide the surgeon to a predefined location in 3D space and allow for specific trajectories, the applications of RA spine surgery will extend beyond the placement of pedicle screws in the near future. Use of the RA method has allowed for iliac fixation in destructive sacral lesions, sacroiliac fusions, and percLIF through Kambin’s Triangle. In addition to improving safety and accuracy, RA technology has allowed for faster recovery, same day discharges, minimal blood loss obviating transfusions, decreased radiation exposure, and shorter lengths of stay (16, 37).

A similar history can be traced back to the introduction of the da Vinci robot® (Intuitive Surgical; Sunnyvale, California). The first ever use of the robot was in 1997 where it assisted during a cholecystectomy. However, the skepticism of the da Vinci robot was prominent at the start of its widespread usage. Doctors complained of learning a brand-new propriety software, patients filed lawsuits for medical damages that occurred during surgeries, and hospitals were averse to the extremely high equipment prices. Slowly, as companies improved both the user interface and hardware of each product, there was increased acceptance of this technology. In 2000, the FDA approved the usage of the da Vinci robot for general laparoscopic surgeries (38). These procedures led to less blood loss, less need for blood transfusion, lower mean pain score, and shorter LOS when compared to patients undergoing the comparable open procedure (39). Just like with the da Vinci robot, the new RA techniques in spine surgery are following a similar path in their technological life cycle.

In the percLIF application, for example, we acknowledge that the technique itself is only semi-autonomous, which further indicates that the future applications for RA spine surgery should revolve around the autonomous performance of surgical actions rather than robotic localization. This will depend on research that provides haptic feedback, feedback loops, and greater robotic arm precision to perform the operator actions that spine surgery requires. This also introduces the potential for the robot to have an MIS retractor tube to dock through the system itself to provide robotic assistance in resection capabilities beyond simple localization. Recently, researchers have examined robotic lumbar facet decortication. Utilizing a preoperative plan similar to that used in percLIF, the robotic arm swings into position. Rather than placing a pedicle screw or tap, a large burr is inserted through the guide to facilitate facet decortication (40, 41). Yet another advancement was shown in the cases of iliac fixation, where we were able to successfully perform iliolumbar fixation percutaneously without the need for a side connector, which is a potential place of weakness and failure (42). Of note, this technique has been implemented by various other groups in the literature and is currently expanding its usage (43–45). Likewise, in the case of sacroiliac fusion, the robot’s ability to accurately place instrumentation without soft tissue destruction is an advantage compared to traditional methods.

As discussed throughout this paper, there are a multitude of advantages to using RA technology in the realm of spine surgery. However, it remains important to also discuss the potential disadvantages as we use the robot for increased indications. The most common of these being the increased cost and subsequent decreased accessibility. These technologies are undoubtedly more expensive than the prior standard navigation platforms, but over the past few years, mass production has cut costs nearly in half as seen with the recently launched third generation Mazor X™ system (46). In terms of cost-effectiveness, there continues to be a lack of broad studies on this topic. Having said that, researchers have started comparing RA to standard technology in key variables that make up the cost-effective category including fluoroscopy time, revision rate, operative room time, and LOS. Fiani et al. reported in their systematic review paper that RA technology improved cost-effectiveness in all of these key subcomponents (46).

Further evaluating the safety, efficiency, cost, and learning curve timeline are all crucial for understanding the true benefits of RA technology versus standard navigation tools. Some of the challenges to proving these benefits include the small sample sizes of previous works thereby making randomized control studies difficult to create (47). Additionally, it is also important to look at the horizon of RA technology - augmented reality (AR). Only a few studies have examined its use in a patient model due to its higher costs and steep learning curve (48, 49). On the other hand, AR has the potential to solve a variety of the current limitations with both RA and traditional navigation technologies including line-of-sight errors, an external camera system, and surgeon attention shift (48, 50, 51). These future innovations will most likely follow the same path RA has: inception, growth, hesitance, and incremental improvement towards large-scale implementation.

## Conclusions

With constant advancements in imaging, navigation, and robotics, surgeons now have even more access to tools that can improve preoperative planning, intraoperative visualization, and postoperative outcomes. Early work in spine surgeries has shown the possible applications of RA procedures beyond pedicle screw placement, but as noted in each application, further studies are needed to demonstrate the long-term clinical benefit for widespread adoption. There are numerous obstacles with integrating new technologies in the operating room including cost, learning curves, and general hesitance towards new methodologies. To begin chipping away at this tentative opinion regarding robotics, more work will need to be done on larger patient populations to continue optimizing the safety and accuracy of robotic-assisted spine surgery.
